# Contextual Fine-Tuning of Language Models with Classifier-Driven Content Moderation for Text Generation

**DOI:** 10.3390/e26121114

**Published:** 2024-12-20

**Authors:** Matan Punnaivanam, Palani Velvizhy

**Affiliations:** Department of Computer Science and Engineering, College of Engineering Guindy, Anna University, Chennai 600025, India; velvizhyp@annauniv.edu

**Keywords:** large language models (LLMs), supervised fine tuning, text classification, BERT, generative models, deep learning, natural language processing (NLP)

## Abstract

In today’s digital age, ensuring the appropriateness of content for children is crucial for their cognitive and emotional development. The rise of automated text generation technologies, such as Large Language Models like LLaMA, Mistral, and Zephyr, has created a pressing need for effective tools to filter and classify suitable content. However, the existing methods often fail to effectively address the intricate details and unique characteristics of children’s literature. This study aims to bridge this gap by developing a robust framework that utilizes fine-tuned language models, classification techniques, and contextual story generation to generate and classify children’s stories based on their suitability. Employing a combination of fine-tuning techniques on models such as LLaMA, Mistral, and Zephyr, alongside a BERT-based classifier, we evaluated the generated stories against established metrics like ROUGE, METEOR, and BERT Scores. The fine-tuned Mistral-7B model achieved a ROUGE-1 score of 0.4785, significantly higher than the base model’s 0.3185, while Zephyr-7B-Beta achieved a METEOR score of 0.4154 compared to its base counterpart’s score of 0.3602. The results indicated that the fine-tuned models outperformed base models, generating content more aligned with human standards. Moreover, the BERT Classifier exhibited high precision (0.95) and recall (0.97) for identifying unsuitable content, further enhancing the reliability of content classification. These findings highlight the potential of advanced language models in generating age-appropriate stories and enhancing content moderation strategies. This research has broader implications for educational technology, content curation, and parental control systems, offering a scalable approach to ensuring children’s exposure to safe and enriching narratives.

## 1. Introduction

Natural language processing (NLP) has rapidly evolved over the past few decades, revolutionizing how machines interact with human language. At its core, NLP involves the ability of machines to understand, interpret, generate, and respond to human language in a meaningful way. This field encompasses various applications such as machine translation, sentiment analysis, text summarization, question answering, and more. With the advent of deep learning and large-scale datasets, NLP models have become more sophisticated and capable of performing tasks that previously required human intervention.

**Figure 1:**
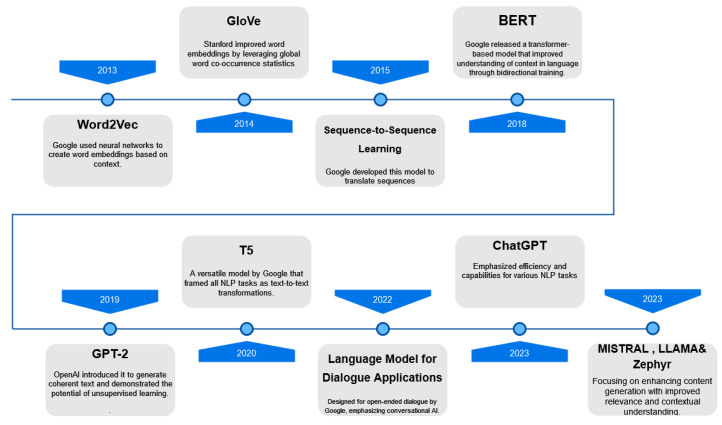
Evolution of LLMs.

The advent of Large Language Models (LLMs) (Figure 1), such as GPT-3 [1],  LLaMA [2], Mistral [3], Zephyr [4], and GEMMA [5]), has significantly accelerated progress in NLP. These models have achieved remarkable success in generating coherent, contextually aware, and meaningful text. However, the generation of domain-specific or context-sensitive content, such as child-friendly stories, remains a challenging task. This challenge arises due to the complexity of maintaining fluency while adhering to specific content guidelines, such as avoiding sensitive or inappropriate themes for children.

Child-friendly content generation requires adherence to multiple constraints, including language simplicity, appropriate subject matter, and creativity suitable for young audiences. While general-purpose language models have shown considerable progress in generating human-like text, they are not always equipped to handle these variation requirements. For example, many models may generate complex vocabulary or introduce themes inappropriate for children. Inappropriate content for children’s stories includes elements like violence and gore, horror themes, inappropriate language, mature themes, moral ambiguity, overly complex narratives, disturbing imagery or scenarios, the absence of positive role models, excessive sadness or despair, and cultural or social insensitivity. These aspects fail to align with the developmental needs, emotional safety, and learning goals appropriate for children’s storytelling. This calls for specialized fine-tuning techniques to ensure that language models can produce text that meets the specific requirements of children’s literature (An example in Box 1).

Box 1 **A Sample Unsuitable Story.**

Let us consider an example story titled—**The Whispering Woods**: Once upon a time, in a small village, there was a forest everyone called The Whispering Woods. The villagers warned children never to go there after sunset because strange sounds were said to echo through the trees—soft whispers that seemed to follow anyone who entered.

Emma, a curious ten-year-old, loved adventure. She had heard stories about the woods but didn’t believe in the whispers. “It’s just the wind”, she thought. One evening, as the sun dipped below the horizon, she decided to prove her bravery.

With her flashlight in hand, Emma crept into the woods. At first, everything seemed normal. The trees stood tall and silent, their branches forming a dark canopy above her. But then, as she ventured deeper, she heard it—a faint whisper.

“Emma…” the voice called softly.

She froze. “Who’s there?” she called out, her voice trembling. But there was no reply. Her flashlight flickered, casting eerie shadows that danced on the trees. Her heart pounded as she turned in circles, trying to find the source of the sound.

The whispers grew louder, and Emma thought she saw a shadow dart behind a tree. Fear gripped her. Suddenly, she remembered the villagers’ warnings. She tried to retrace her steps, but the path seemed different now, twisted and unfamiliar.

Just when she thought she was hopelessly lost, she heard another voice—this one warm and familiar. “Emma! Are you there?” It was her father, who had come searching for her. Relief washed over her as he emerged with a lantern.

The whispers faded as they hurried out of the woods together. At the edge of the forest, Emma turned back for one last look and saw the trees sway gently as if bidding her goodbye.

From that day on, Emma learned to heed the warnings of those who cared for her. She realized that bravery wasn’t just about facing fears but also about knowing when to listen.

**The above story is an inappropriate one because it includes elements that can induce fear and anxiety, such as mysterious whispers, shadowy figures, and a young child getting lost in an ominous forest. For a story to be suitable for children, it should prioritize reassurance and clear resolutions.**

**Figure 2:**
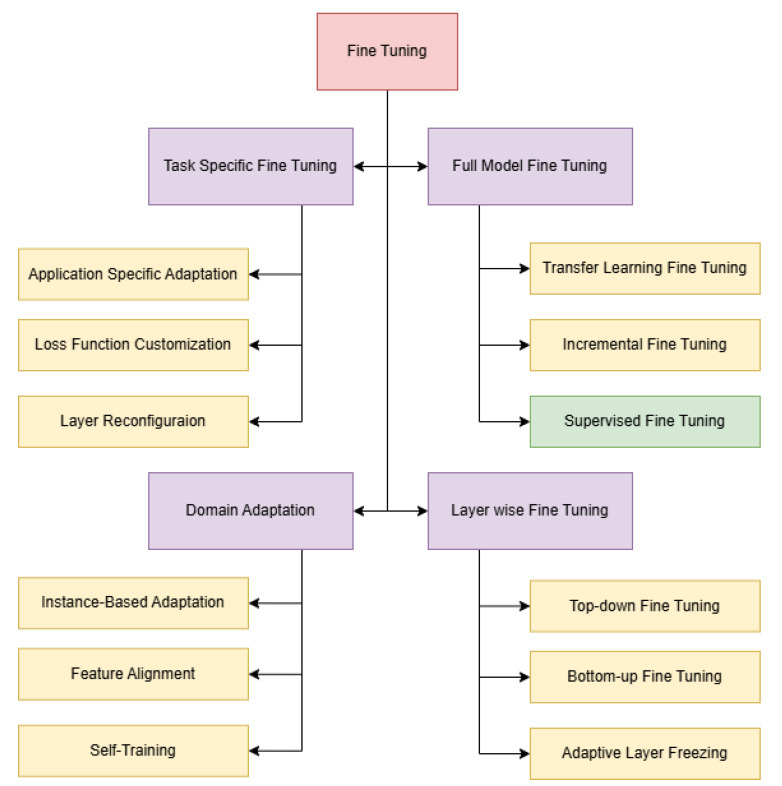
Types of fine-tuning.

To address this, Supervised Fine-Tuning (SFT) (Figure 2) [6,7,8] has emerged as a powerful technique to customize Large Language Models for specialized tasks. By training a model on a dataset of examples specific to a given domain, SFT enables the model to generate content that aligns closely with the desired task or objective. In this case, fine-tuning can help guide models to generate stories that are not only coherent but also child appropriate, avoiding themes that may be too complex or unsuitable.

To further ensure the generation of child-appropriate content, we propose the integration of a classifier model within the storytelling pipeline. This classifier is designed to evaluate the suitability of individual story segments, assessing whether they align with predefined guidelines for children’s literature. Specifically, the classifier flags portions of text that may contain inappropriate themes, such as violence or complex vocabulary, marking them as “unsuitable”. When such a section is identified, it is passed back to the fine-tuned Large Language Model (LLM) for reframing, ensuring that the content is revised to meet the necessary child-friendly standards while maintaining narrative coherence. This additional layer of content moderation helps ensure that the final output is engaging, appropriate, and sensitive to children.

### 1.1. Child-Friendly Story Generation with Classifier Integration

In recent years, generating child-friendly stories using deep learning models has gained traction due to its potential in education, entertainment, and literacy development. These models are being designed to automate story creation that is engaging and safe for young readers. A core challenge, however, is ensuring that the generated stories align with children’s cognitive and emotional development. This means that the text should be simple enough for a child to comprehend and avoid themes that are inappropriate for young audiences. The primary focus of this study is to fine-tune large-scale language models, such as Zephyr-7B, Mistral-7B, and LLaMA2 7B, to generate stories suitable for children. While these models have demonstrated notable success in general text generation, they require additional tuning to ensure that the generated content adheres to specific child-appropriate standards. To further enhance the child-friendliness of the generated content, a classifier model is integrated into the system. The classifier is designed to evaluate story segments and determine whether they are suitable or unsuitable for children. If any portion of the story is flagged as inappropriate, it is passed through the fine-tuned Large Language Model (LLM) for reframing. This ensures that the unsuitable part is reworded in a child-appropriate manner while maintaining the overall coherence of the story. The classifier acts as an essential safeguard, ensuring that the final story output meets the necessary standards of simplicity, age appropriateness, and emotional sensitivity.

This two-stage approach—first classifying the content and then refining it when necessary—provides an additional layer of control, enabling the generation of stories that are not only coherent but also safe for young readers.

### 1.2. Fine-Tuning Large Language Models for Child-Friendly Story Generation

In this article, we compare three state-of-the-art model—Zephyr-7B, Mistral-7B, and LLaMA2 7B—to assess their performance in generating child-friendly stories. These models represent the latest advancements in transformer-based architectures and have proven their ability to generate high-quality text in various domains. However, the task of generating content for children introduces unique constraints that must be handled through Supervised Fine-Tuning. We propose using a custom dataset of 515 stories specifically curated for children’s literature. Each story in the dataset is paired with a title and a prompt, guiding the model in generating contextually relevant and child-appropriate stories. The use of Supervised Fine-Tuning (SFT) will enable the models to learn from this dataset and adjust their generation patterns to align with the characteristics required for children’s content.

### 1.3. Contributions of the Proposed Research

The key contributions of this research are as follows:

1. Custom Dataset Creation: We have developed a dataset comprising 515 stories explicitly designed for children, including titles and prompts to guide story generation. The dataset ensures the model is trained to produce age-appropriate content that meets children’s cognitive and emotional needs.
2. Supervised Fine-Tuning of Multiple Models: We have applied Supervised Fine-Tuning (SFT) to three state-of-the-art language models—Zephyr-7B, Mistral-7B, and Llama2 7B—enabling them to generate child-friendly stories. The fine-tuning process allows these models to adapt to the unique requirements of children’s literature, ensuring simplicity, thematic appropriateness, and creativity.
3. Classifier Model for Content Evaluation: A novel classifier model is introduced, which evaluates the generated stories by flagging parts of the content that may be inappropriate or unsuitable for children. Once flagged, these portions are passed through the fine-tuned LLM for automatic reframing, ensuring the entire story is coherent and aligned with child-friendly standards. This addition provides a second layer of control in maintaining the quality and appropriateness of the generated text.
4. Comprehensive Comparative Analysis: We conducted a comparative analysis of three models by evaluating their performance across critical aspects, such as simplicity, and content appropriateness. This evaluation aimed to assess how well each model handles child-friendly story generation. To further ensure the quality and safety of the content, we incorporated human evaluation. A group of PhD scholars from a state university manually assessed the stories generated by each model based on five key criteria: Language Simplicity and Clarity, Suitability for Children, Deceptiveness, Story Structure and Pacing, and Creativity and Originality. To ensure consistency, we calculated the Inter-Annotator Agreement (IAA) using the Kappa score.
5. Evaluation of Generated Stories: In addition to standard NLP metrics such as ROUGE, METEOR, and BERT Score, we incorporate human evaluations to assess the suitability of the generated stories for children. This includes factors such as creativity, thematic appropriateness, readability, and emotional tone, ensuring the final content is engaging and suitable for young readers.

Through these contributions, we aim to bridge the gap between general-purpose language models and the specific requirements of child-friendly story generation. By integrating fine-tuning techniques and a classifier for content verification, we enhance the ability of Large Language Models to generate high-quality, child-appropriate stories, while also providing valuable comparative insights into the efficacy of different model architectures for this specialized task.

## 2. Literature Survey

The rapid development of LLMs, such as BERT, GPT, and their fine-tuned versions, has significantly improved the ability to generate coherent and contextually rich text. However, the suitability of these models for generating content specifically tailored to children’s cognitive and emotional needs remains underexplored.

This literature survey focuses on three core aspects of the research: the evolution of LLMs and their role in text generation, the fine-tuning processes that enhance their performance for specialized tasks, and the integration of classifier models to filter and evaluate the generated content based on suitability. By examining prior work in these areas, we aim to highlight the limitations of existing models in children’s content generation and explore how the combination of LLM fine-tuning and classifier integration can address these gaps. Ultimately, this survey provides the contextual background for our proposed approach, which builds upon and extends prior research to create a more robust framework for generating suitable content for young audiences.

In the domain of Large Language Models (LLMs) and content generation, we started with early models like Word2Vec [9] and GloVe [10], which introduced word embeddings based on semantic similarities. These models enabled basic content generation but struggled with maintaining long-range dependencies, especially when the text required coherence over multiple sentences or when tailored content, such as children’s literature, was needed [11]. The transition to transformer-based architectures like BERT [12],  GPT-3 [1], and T5 [13] revolutionized content generation and natural language understanding, bringing substantial improvements in contextual comprehension and   fluency [13,14]. However, even these sophisticated models demonstrated limitations in generating content for specific, sensitive audiences, such as children, where appropriateness and simplicity are key [15].

The integration of classifier models for moderating generated content has gained traction in recent years, primarily in areas like hate speech detection [16] and misinformation filtering [17]. These classifiers evaluate and filter content to ensure it aligns with specific ethical or appropriateness standards, though research on applying such technologies to children’s content remains sparse. This study aims to bridge this gap by focusing on fine-tuning LLMs for children’s content generation [18], while also incorporating classifier models to assess content suitability, a direction previously underexplored [1,12].

Key themes examined in this review include early language models, transformer-based models for content generation, fine-tuning methodologies, and the role of classifier models in moderating generated content. By mapping the evolution of these technologies, it becomes possible to delineate how current advancements can be leveraged to produce content specifically tailored to sensitive audiences like children.

### 2.1. Thematic Categorization

The section explores the evolution of Large Language Models (LLMs) and their growing capabilities in natural language generation. It examines the progression from early rule-based systems to advanced transformer-based architectures like GPT, BERT, LLaMA, Mistral, Zephyr, etc., which have revolutionized contextual understanding and content generation. Fine-tuning strategies have been emphasized for adapting pre-trained models to specific domains, such as children’s literature. The section also surveys classifier models that are critical for content moderation and categorization, enabling automated systems to distinguish appropriate themes for different audiences. Evaluation metrics like ROUGE, METEOR, and BERT Score are reviewed to assess the generated content’s quality and relevance. This thematic framework combines the generative strengths of LLMs with the classifiers’ precision to effectively categorize and curate content for children’s developmental needs.

#### 2.1.1. Early Language Models and Content Generation

The foundation of automated content generation was laid by early language models such as Word2Vec and GloVe, which represented words as continuous vectors based on their semantic relations in large corpora [9,10]. These models enabled basic sentence-level content generation and served as the backbone for tasks such as sentiment analysis and machine translation [19]. Sequence-to-sequence (Seq2Seq) models, such as the one introduced by [20], further pushed the envelope by allowing end-to-end neural networks to translate entire sequences of words into another sequence, expanding possibilities for more complex text generation.

Despite their initial success, these models exhibited several limitations, particularly in managing long-range dependencies and ensuring context coherence across multiple sentences. This challenge was amplified when generating content for specific target audiences, such as children, where not only coherence but also simplicity and appropriateness are crucial [21]. As a result, more sophisticated models such as transformers were developed to address these deficiencies.

#### 2.1.2. Transformer-Based Models for Language Generation

The development of transformer-based models fundamentally changed the landscape of Natural Language Processing (NLP). The transformer architecture, introduced by [14], outperformed traditional models by enabling the parallel processing of input sequences, allowing it to handle long-range dependencies with much greater efficiency. Models like BERT (Bidirectional Encoder Representations from Transformers) and GPT-3 (Generative Pre-trained Transformer) represent the cutting edge in NLP [15]. These models demonstrate significant advancements in tasks ranging from question answering to text summarization and story generation [1,13].

However, while transformer-based models excel in generating rich and coherent content, applying them to children’s content generation remains a challenge. These models, when used out-of-the-box, often generate content that is too complex or inappropriate for younger audiences. For instance, ref. [22] highlighted how generated texts from models like GPT-3 can veer into unsuitable themes if not carefully monitored. This gap signals the need for specialized fine-tuning and content moderation strategies to ensure that the generated material aligns with the cognitive and developmental needs of children.

#### 2.1.3. Fine-Tuning Large Language Models (LLMs)

Fine-tuning pre-trained LLMs has proven essential for adapting models to specific domains or tasks. For example, [22] demonstrated that fine-tuning BERT for sentiment analysis significantly improves its performance in that domain. Similarly, [1] showcased the ability of GPT-3 to adapt to various creative writing tasks through fine-tuning. This process allows the model to become more sensitive to context, enhancing generation accuracy and ensuring that the content aligns with domain-specific requirements [23].

Fine-tuning is particularly critical when generating content for sensitive audiences, such as children. Mistral [3] and Zephyr [4] are recent models that have been fine-tuned for domain-specific applications, including technical writing [24]. However, the fine-tuning of LLMs specifically for generating children’s content remains underexplored in the literature. Tailoring LLMs for this purpose, by training them on datasets that focus on age-appropriate language and themes, offers a unique opportunity to address this gap and ensure that generated content is suitable for young readers.

#### 2.1.4. Classifier Models in Content Moderation

The application of classifier models in content moderation [25] is a rapidly growing area of research, particularly in light of the increasing volume of user-generated content online. Classifiers such as BERT-based models and traditional techniques like Random Forest have been effectively deployed to detect offensive language, hate speech, and misinformation [26]. These models analyze generated content and filter out inappropriate material based on predefined ethical standards.

Despite the success of classifiers in moderating content for adult audiences, there is a notable lack of research on their application to children’s content generation. Refs. [21,25] primarily focused on detecting harmful content but did not consider the content suitability for younger audiences. To address this gap, the integration of classifiers capable of evaluating generated text for age appropriateness and thematic suitability is essential.

#### 2.1.5. Evaluation Metrics in Content Generation

Evaluating the quality of generated content requires robust and reliable metrics. Standard evaluation metrics such as ROUGE, METEOR, and BERT Score are frequently employed to assess the fluency, coherence, and relevance of generated text [27,28,29]. These metrics have become the gold standard in evaluating model performance across tasks like text summarization and machine translation. However, while these metrics are useful for general text generation tasks, they fall short in evaluating content intended for specific audiences like children. The current evaluation methodologies do not take into account the developmental and cognitive requirements of younger readers. Thus, developing more refined evaluation techniques that address the unique needs of children’s literature is critical for advancing research in this area.

### 2.2. Comparative Analysis

In synthesizing the literature, it becomes clear that substantial progress has been made in the areas of content generation, model fine-tuning, and content moderation. However, the application of these advancements to children’s content generation remains limited. The development of transformer-based models such as BERT and GPT-3 revolutionized automated content generation, providing sophisticated tools for producing coherent, contextually rich text [14,15]. Yet, these models require careful fine-tuning and monitoring to generate content suitable for sensitive audiences, such as children [25].

The literature reveals a clear gap in integrating fine-tuned LLMs with classifier models specifically designed to assess content for child-appropriateness. While classifiers have been employed for general content moderation [26,30], their application to children’s literature remains underexplored. This research aims to address that gap by developing a novel approach that combines fine-tuning LLMs with classifier models to generate high-quality, child-appropriate content, an area that has been largely overlooked in  previous studies.

The advancements made through fine-tuning techniques and content moderation using classifier models have been highlighted. However, significant gaps remain in ensuring that generated content is tailored to specific audiences, particularly children.

By building upon the existing body of knowledge, this research seeks to fill these gaps by developing methods for fine-tuning LLMs specifically for generating child-appropriate content and incorporating classifier models to assess suitability. This approach will offer a novel contribution to the field by addressing the critical need for high-quality, child-friendly content generation.

**Figure 3:**
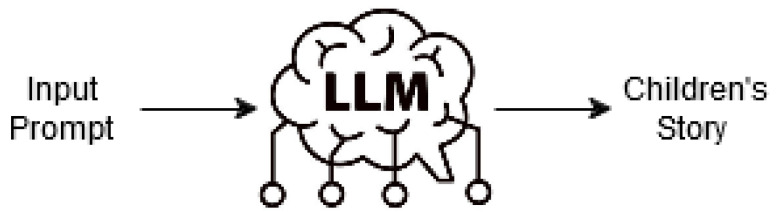
Traditional story generation using LLM.

**Figure 4:**
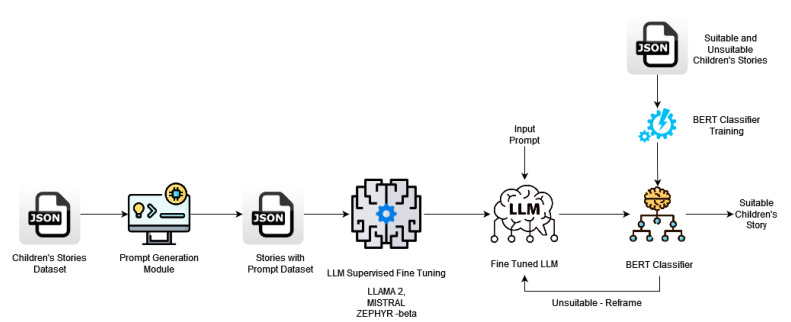
Pipeline of the proposed system.

The evolution of story generation techniques can be observed through the contrast between the traditional approach (Figure 3) and the proposed framework (Figure 4). Traditional methods primarily involve indirect workflows. For instance, prompts are crafted based on predefined rules or templates, and generated outputs often undergo further manual refinement to ensure relevance and suitability. This process is both time-consuming and limited in its scalability due to dependency on human expertise.

In contrast, the proposed framework streamlines the generation process by leveraging advanced fine-tuned Large Language Models (LLMs). As illustrated in Figure 4, the proposed method directly integrates domain-specific fine-tuning and classification within the LLM pipeline. This enables the generation of stories that align with appropriateness criteria without requiring intermediate manual adjustments. The addition of classifier-based moderation further ensures that the outputs meet the predefined standards of suitability for children, thereby enhancing efficiency and scalability.

This direct and contextually aware pipeline, unique to the proposed method, represents a significant shift from traditional techniques, offering a more robust and automated solution for generating appropriate content tailored to specific audiences.

## 3. Methodology

This section outlines the detailed steps undertaken to fine-tune and evaluate the three selected models—Zephyr-7B-Beta, Mistral-7B, and Llama2 7B—for the task of generating child-friendly stories (Table 1). The primary goal was to adapt these models to a specialized task, ensuring that the stories generated are not only creative and coherent but also suitable for children, avoiding complex, inappropriate, or violent content (Figure 4). To achieve this, we leveraged a custom dataset consisting of 515 carefully curated stories, each paired with a title and an associated prompt. The methodology is structured to provide clarity on the architecture of the models, the dataset preparation, and the fine-tuning process, and compare their performance.

### 3.1. Mistral-7B

Mistral-7B is a highly optimized language model that contains 7 billion parameters but with architectural tweaks that emphasize speed and efficiency without compromising on quality. The Mistral-7B Model is described as follows:

1. Architecture: Mistral-7B is also based on the transformer model but incorporates enhancements in its attention mechanisms and memory management. These optimizations are designed to make the model more efficient during both training and inference, reducing the computational resources required. The use of improved layer normalization and attention mechanisms allows Mistral-7B to achieve faster convergence during training and faster response times during text generation tasks.
2. Unique Features: One of the key strengths of Mistral-7B is its speed. Mistral-7B is optimized for faster text generation while maintaining a similar level of accuracy and coherence to other models in its parameter range. This is particularly important for real-time applications like chatbot-based storytelling or interactive children’s story platforms, where fast response times are crucial.
3. Use Case: Mistral-7B is well suited for real-time and low-latency applications. Its fast inference times make it ideal for interactive storytelling, where children may interact with a story-generation system in real-time. The model’s training process is designed to minimize latency without sacrificing the creative aspects of story generation.

### 3.2. LLaMA2 7B

LLaMA2 7B is one of the most widely used open-source models in the NLP community for text generation. It is known for its high performance across various text-generation tasks, making it a strong candidate for creative writing, including generating  children’s stories.

1. Architecture: Like the other models, Llama2 7B follows the transformer architecture with 7 billion parameters. It includes several layers of self-attention and feed-forward networks, and it is optimized for large-scale text generation and understanding tasks. The model is pre-trained on a massive dataset covering a wide range of domains, allowing it to generalize well across different text-generation tasks.
2. Performance: Llama2 7B is known for its high performance in generating fluent, coherent, and contextually accurate text. Its robust training process enables it to adapt to specific tasks, making it particularly effective in creative writing tasks. The model performs well in generating long-form text that requires maintaining thematic and narrative consistency, which is essential for storytelling.
3. Why it is Widely Used: The popularity of Llama2 7B stems from its open-source nature and strong performance across different tasks. It has been fine-tuned and evaluated on numerous benchmarks, making it a trusted choice for various natural language generation applications. In this study, its adaptability to child-friendly story generation is tested, given its ability to produce high-quality and engaging narratives.

### 3.3. Zephyr-7B-Beta

Zephyr-7B-Beta is a 7-billion-parameter language model built using the transformer architecture, specifically designed for large-scale natural language understanding and generation tasks.

1. Architecture: Like most Large Language Models, Zephyr-7B-Beta employs the transformer architecture, consisting of multiple layers of self-attention and feed-forward networks. Each layer contains several attention heads, allowing the model to capture long-range dependencies in the text. This architecture is well suited for tasks that require coherent and contextually appropriate text generation over extended passages.
2. Size: With 7 billion parameters, Zephyr-7B-Beta strikes a balance between model complexity and computational efficiency. It is large enough to perform exceptionally well on text generation tasks while being small enough to be fine-tuned and deployed on consumer-grade hardware or cloud services.
3. Intended Use Case: Zephyr-7B-Beta is designed for general-purpose text generation tasks, such as dialogue generation, summarization, and creative writing. Pre-trained on diverse datasets, it is capable of generating high-quality text across various domains. For this study, Zephyr-7B-Beta is fine-tuned to generate child-friendly stories, focusing on simplicity, engagement, and appropriate language for young audiences.

### 3.4. Dataset Preparation

The dataset used for this study consists of a custom-built collection of 515 stories, each paired with a title and a corresponding prompt. These stories were handpicked one by one for their suitability for children and sourced from various platforms, including Kaggle, GitHub, and the web [31,32,33].

To ensure cultural diversity and broad applicability, the dataset includes narratives originating from diverse cultural traditions. This inherent cultural richness in the dataset ensures that the fine-tuned models have a foundational understanding of diverse ethical and cultural contexts. It enables the generation of stories that are both contextually relevant and respectful of different cultural norms, fostering inclusivity in the generated content.

The preparation of this dataset involved several key steps.

**Figure 5:**
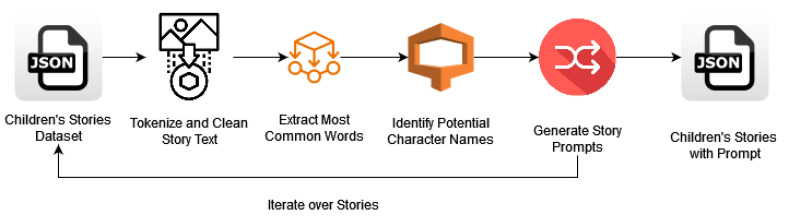
Prompt generation module.

1. Story and Title Compilation: Initially, the stories and their titles were gathered and structured in a JSON file format. Each entry in this file includes the full text of a story along with its title, forming the foundational dataset for the subsequent processing steps.
2. Preparing Prompts for Child-Friendly Stories: In the process of adapting generative models for producing child-friendly stories, a key step involved crafting detailed prompts (Algorithm 1) that would guide the models in generating coherent and engaging narratives (Figure 5). This was achieved through a systematic approach involving text extraction and prompt generation based on the characteristics of each story. The following sections detail how this was accomplished using Python code and the Natural Language Toolkit (NLTK).

**Algorithm 1** Prompt generation process.
  1:**Input:** Stories from input file  2:**Output:** Processed stories with generated prompts  3:
**Text Extraction:**
  4:Extract key details from each story;  5:Tokenization: Split text into words, remove stopwords;  6:Frequency Analysis: Identify common words and themes;  7:Character Extraction: Use regex to find capitalized names;  8:Sentence Extraction: Extract first and last sentences;  9:
**Prompt Generation:**
10:Use extracted elements to create prompts;11:Random Sampling: Select up to 5 themes and 3 character names;12:Template Usage: Apply various storytelling templates;13:
**Implementation:**
14:Read stories from input file (various text formats);15:Generate unique prompts for each story;16:Append prompts to story objects;17:Save updated stories to output file in JSON format;18:Create final dataset with titles and prompts in new JSON file;


### 3.5. Key Components of the Prompt Generation Process

1. Text Extraction: The process begins by extracting essential elements from each story using a custom function designed to capture the key details. This involves several important steps:
  (a) Tokenization and Stopword Removal: The text is split into individual words, and common stopwords (e.g., “the”, “and”, and “is”) are filtered out to retain only meaningful terms.
  (b) Frequency Distribution Analysis: A frequency analysis is conducted to identify the most common words, revealing key themes within the story.
  (c) Character Name Extraction: Character names are identified using regular expressions that capture capitalized words, with a limit set on the number of names extracted.
  (d) Sentence Extraction: The first and last sentences of the story are extracted to serve as reference points for prompt generation.
2. Detailed Prompt Generation: The next step involves using the extracted elements to create rich, context-aware prompts. This is achieved by a function that generates prompts based on predefined templates to encourage narrative depth. The process includes the following:
  (a) Random Sampling: Key themes and character names are randomly selected to diversify the prompts, choosing up to five themes and three characters for each prompt.
  (b) Template Usage: A variety of storytelling templates are employed, each designed to generate unique narrative styles.
3. Implementation Process: The entire prompt generation is managed by a central function that processes the stories and generates the prompts, with the following stages:
  (a) File Handling: The stories are read from an input file, ensuring compatibility with various text formats.
  (b) Prompt Assignment: A unique prompt is generated for each story and appended to the story object.
  (c) Output Generation: The updated stories, now containing their prompts, are saved to an output file in JSON format.
4. Final Dataset Creation: The processed stories, complete with titles and generated prompts, are saved into a new JSON file. This organized dataset forms the basis for fine-tuning the language models used in this study.

### 3.6. Dataset Description: Classifer Model

The dataset employed in this study comprises a carefully curated collection of  500 stories, each accompanied by a title. The primary objective of this dataset is to train a classifier model that can categorize stories as either suitable or unsuitable for children.

**Dataset Structure:** Each entry in the dataset (Box 2) consists of two main components:

Title: A brief, descriptive title reflecting the main theme of the story.

Story: The full text of the story, crafted to engage young readers while avoiding themes that may be inappropriate for children.

Box 2 **Dataset Structure of Classifier Model.**

DatasetDict({

train: Dataset({

features: [‘title’, ‘story’],

num_rows: 500

})

})

{‘title’: Value(dtype = ‘string’, id = None), ‘story’: Value(dtype = ‘string’, id = None)}

### 3.7. Fine-Tuning Process: LLaMA 2, Mistral-7B, and Zephyr-7B-Beta

**Figure 6:**
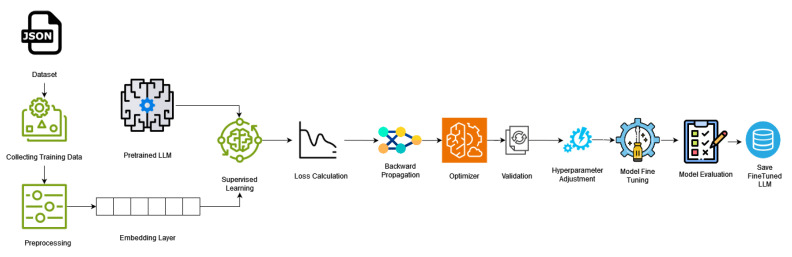
Supervised fine-tuning.

The fine-tuning process (Figure 6) for the LLaMA 2, Mistral-7B, and Zephyr-7B-Beta models (Algorithm 2) was conducted to adapt these powerful generative models for generating child-friendly stories. While these models are highly capable in general text generation tasks, they required specialized fine-tuning to ensure that the output was appropriate for children. A Supervised Fine-Tuning (SFT) approach was used, along with a custom dataset of 515 child-friendly stories. The primary objective was to minimize the difference between the model’s generated text and the reference stories, ensuring fluency, coherence, and suitability for a younger audience.

#### 3.7.1. Training Setup

The fine-tuning process for all three models (LLaMA 2, Mistral-7B, and Zephyr-7B-Beta) was conducted using the SFT Trainer. Given the substantial number of parameters (Table 2) in these models, the fine-tuning was computationally intensive. To manage this, we employed Parameter-Efficient Fine-Tuning (PEFT) techniques such as Low-Rank Adaptation (LoRA), which focused on updating specific components within the attention layers. This allowed for fine-tuning without retraining the entire model, significantly reducing memory and computational requirements while preserving performance.

To further optimize resource usage, mixed precision training was utilized. This approach leverages libraries such as Hugging Face’s Accelerate to improve training speed and decrease memory overhead. By employing these techniques, we ensured the efficient use of computational resources without compromising the quality of fine-tuning.

All training was performed on a single Nvidia Quadro P5000 GPU, highlighting the framework’s ability to handle resource-intensive tasks in cost-effective, single-GPU setups. This demonstrates the scalability and practical feasibility of our approach, making it accessible for deployments with constrained hardware resources.

- Model: Each model, LLaMA 2, Mistral-7B, and Zephyr-7B-Beta, is a large-scale generative language model capable of handling various natural language generation tasks. For this research, LoRA was applied to the attention layers of the models, allowing efficient fine-tuning with minimal memory requirements.
- Training Dataset: The same custom dataset, consisting of 515 child-friendly stories, was used for all models. Each story in the dataset had an associated prompt and title. The dataset was tokenized using each model’s pre-trained tokenizer, resized to accommodate the unique vocabulary of the child-friendly dataset, ensuring proper tokenization and interpretation during the training process.
- Gradient Checkpointing: To further manage memory usage, gradient checkpointing was enabled. This technique reduces memory consumption by recomputing intermediate activations during backpropagation, allowing the models to handle longer sequences without exceeding memory limitations.

#### 3.7.2. Training Arguments

The training configuration for all three models shared similar setups, fine-tuned for performance optimization:

- Batch Size: Due to memory constraints, the batch size was set to 1 per device for both training and evaluation.
- Learning Rate: A fixed learning rate of $1\times 10^{-5}$ was employed for stable convergence.
- Gradient Accumulation Steps: Set to 4 to allow gradient accumulation over multiple steps, effectively addressing the small batch size limitation.
- Evaluation Strategy: Each model was evaluated after each epoch on a validation set to monitor performance and avoid overfitting.
- Number of Epochs: All models were fine-tuned for 6 epochs, with early stopping based on validation loss.

#### 3.7.3. Loss Function

For all three models, Cross-Entropy Loss (Equation (1)) was used during fine-tuning. This loss function is suitable for text generation tasks, as it measures the divergence between the predicted token distribution and the actual token distribution in the target sequence. By minimizing this loss, the models were guided to generate text that closely resembled the reference child-friendly stories.

The Cross-Entropy Loss *L* is defined as follows:

(1) $$L=-\frac{1}{N}\sum_{i=1}^{N}\left(y_{i}\log\left(p_{i}\right)+\left(1-y_{i}\right)\log\left(1-p_{i}\right)\right)$$

In Equation (1), *L* represents the Cross-Entropy Loss, and *N* denotes the number of samples. $y_{i}$ is the true label for the *i*-th sample, where it takes a value of either 0 or 1, while $p_{i}$ is the predicted probability for the positive class (label 1) for the *i*-th sample. The term log stands for the natural logarithm.

In this formula, we have the following:

- The first term $y_{i}\log\left(p_{i}\right)$ contributes to the loss when the true label $y_{i}$ is 1, which means that the model should predict a high probability $p_{i}$ for the positive class.
- The second term $\left(1-y_{i}\right)\log\left(1-p_{i}\right)$ contributes to the loss when the true label $y_{i}$ is 0, indicating that the model should predict a low probability $p_{i}$ for the positive class.
- The overall loss is averaged over all samples to provide a measure of how well the model is performing across the entire dataset.

#### 3.7.4. Optimizer and Learning Rate Scheduler

The AdamW Optimizer (Equation (5)) was used for fine-tuning all three models. AdamW decouples weight decay from the gradient update process, making it effective for fine-tuning large models. A cosine learning rate schedule with warm-up steps was applied, gradually increasing the learning rate in the initial stages of training, followed by a slow decay, preventing large fluctuations during optimization.

The AdamW optimizer is an extension of the Adam optimizer that includes weight decay (L2 regularization). It is defined as follows:

The AdamW update rule is as follows:

1. Update biased first moment estimate $m_{t}$:

(2) $$m_{t}=\beta_{1}m_{t-1}+\left(1-\beta_{1}\right)g_{t}$$

In Equation (2), $m_{t}$ represents the biased first moment estimate at time step *t*, while $m_{t-1}$ denotes the first moment estimate from the previous time step t−1. The parameter $\beta_{1}$ is the momentum parameter for the first moment, which is typically set to 0.9, controlling the exponential decay rate. Finally, $g_{t}$ refers to the gradient of the loss function with respect to the parameters at time step *t*.

2. Update biased second moment estimate $v_{t}$:

(3) $$v_{t}=\beta_{2}v_{t-1}+\left(1-\beta_{2}\right)g_{t}^{2}$$

In Equation (3), $v_{t}$ represents the biased second moment estimate at time step *t*, while $v_{t-1}$ refers to the second moment estimate from the previous time step t−1. The parameter $\beta_{2}$ controls the second moment, typically set to 0.999, and is used to adjust the exponential decay rate for the second moment. Additionally, $g_{t}^{2}$ is the square of the gradient of the loss function at time step *t*, which is used to calculate the second moment.

3. Bias correction for $m_{t}$ and $v_{t}$:

(4) $${\hat{m}}_{t}=\frac{m_{t}}{1-\beta_{1}^{t}},\;{\hat{v}}_{t}=\frac{v_{t}}{1-\beta_{2}^{t}}$$

In Equation (4), ${\hat{m}}_{t}$ represents the bias-corrected first moment estimate, while ${\hat{v}}_{t}$ is the bias-corrected second moment estimate. The correction factor for the bias in $m_{t}$ is given by $1-\beta_{1}^{t}$, and the correction factor for the bias in $v_{t}$ is represented by $1-\beta_{2}^{t}$. These correction factors are used to adjust the first and second moment estimates to counteract the bias introduced by initialization.

4. Parameter update with weight decay (regularization term):

(5) $$\theta_{t+1}=\theta_{t}-\eta \left(\frac{{\hat{m}}_{t}}{\sqrt{{\hat{v}}_{t}}+\epsilon }+\lambda \theta_{t}\right)$$

In Equation (5), $\theta_{t+1}$ denotes the updated parameters at the next time step t+1, while $\theta_{t}$ represents the current parameters at time step *t*. The learning rate, denoted by η, scales the update step. A small constant ϵ is added to prevent division by zero, typically set to $10^{-8}$. Finally, λ refers to the weight decay parameter used for regularization.

In summary, these equations define the AdamW optimizer, which combines adaptive learning rates (from Adam) with weight decay (for regularization) to improve model performance during training.

#### 3.7.5. Training Configuration

- Batch Size: Set to 1 per device for both training and evaluation due to memory constraints.
- Learning Rate: Fixed at $1\times 10^{-5}$ to ensure stable convergence.
- Max Token Length: For the models—Mistral-7B and Zephyr-7B-Beta, the maximum sequence length was set to 2048 and for LLaMA 2 the maximum sequence length was set to 1024.
- Epochs: The models were trained for 6 epochs.
- Gradient Accumulation Steps: Set to 4 for all models, allowing for gradient accumulation across multiple steps.
- Evaluation Frequency: The models were evaluated after each epoch.
- Optimizer: AdamW with a cosine learning rate schedule.

#### 3.7.6. Training Process

**Figure 7:**
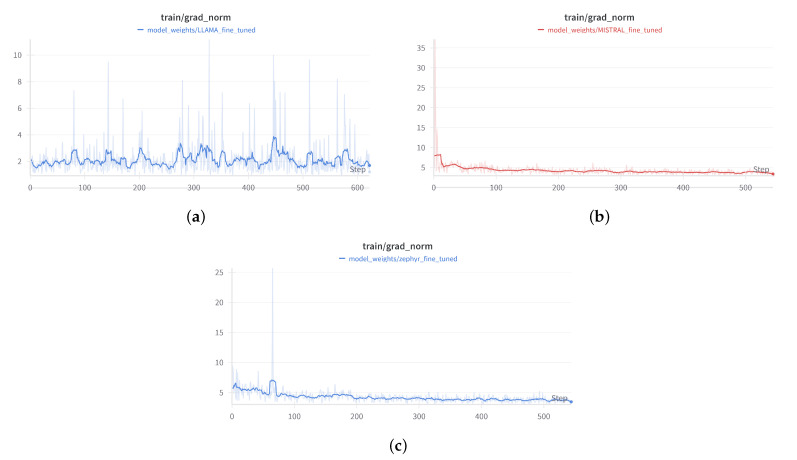
Gradient normalization of LLaMA, Mistral, and Zephyr models. (**a**) LLaMA gradient normalization; (**b**) Mistral gradient normalization; (**c**) Zephyr gradient normalization.

**Figure 8:**
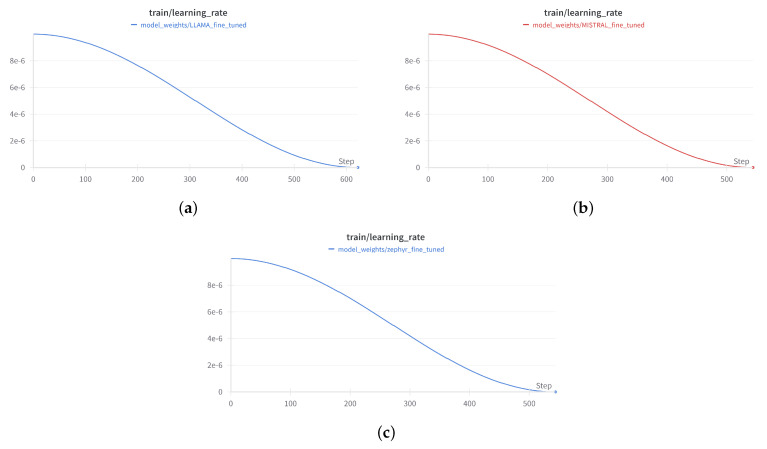
Learning rate of LLaMA, Mistral, and Zephyr models. (**a**) LLaMA learning rate; (**b**) Mistral learning rate; (**c**) Zephyr learning rate.

**Figure 9:**
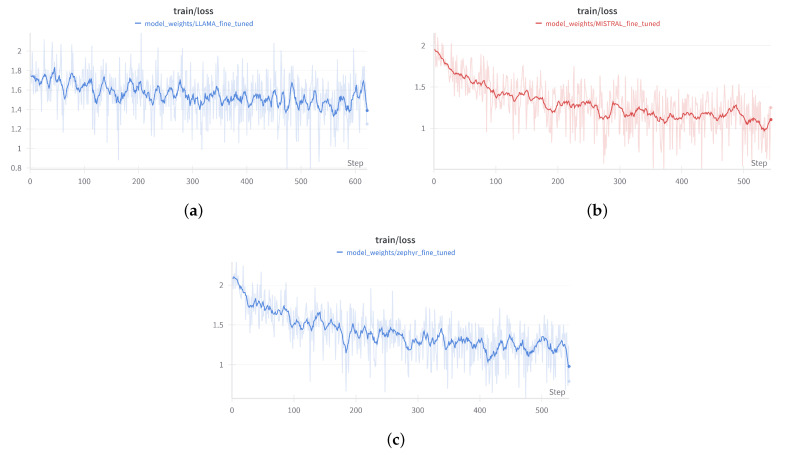
Training loss of LLaMA, Mistral, and Zephyr models. (**a**) LLaMA training loss; (**b**) Mistral training loss; (**c**) Zephyr training loss.

For all three models, the fine-tuning process began with loading the pre-trained model and resizing the tokenizer to fit the custom dataset. LoRA was applied to adapt the attention layers, making the models capable of generating child-friendly stories without retraining the entire network from scratch. Throughout the training process, the models were periodically evaluated (Figure 7, Figure 8 and Figure 9) on a validation dataset to monitor performance and prevent overfitting. Training continued for six epochs, with early stopping based on validation loss to ensure optimal performance and to avoid overfitting the training data.

By using LoRA and gradient checkpointing, the fine-tuning process was completed efficiently, with significantly reduced memory consumption and faster training times for all three models.
**Algorithm 2** Fine Tune Language Model.  1:**Input:** pre_trained_model, children_stories_dataset  2:**Output:** fine_tuned_model  3:Begin  4:Initialize pre-trained model (Mistral, Zephyr, or BERT)  5:Load children_stories_dataset with suitable content  6:Preprocess dataset  7:   Tokenize text data  8:   Normalize text to lower case, remove unwanted symbols  9:   Split data into training and validation sets10:Configure training parameters11:   Set learning rate, batch size, epochs12:   Use early stopping to prevent overfitting13:Fine-tune model14:   Train model on training set15:   Validate on validation set after each epoch16:Evaluate the model using validation loss and metrics (ROUGE, METEOR)17:Save the fine-tuned model for story generation18:End

### 3.8. Design of the LLM Agent

**Figure 10:**
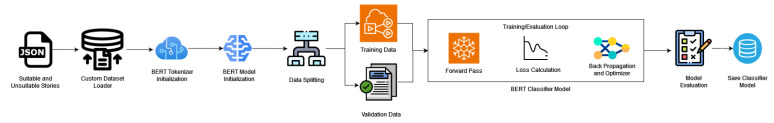
BERT Classifier.

The Large Language Model (LLM) agent is an AI-driven tool designed to generate and refine stories tailored for children. By utilizing advanced natural language processing techniques, the LLM agent produces engaging narratives based on user input, themes, and character details. To ensure that the generated content is appropriate for young readers, a classifier model (Figure 10) is integrated within the agent’s framework. This classifier evaluates the suitability of each generated draft, iteratively refining the output until it meets the established criteria for age-appropriate themes and language. This approach enhances the quality and safety of the stories, making them ideal for educational and entertainment purposes.

In our research framework, the integration of a classifier model with a Large Language Model (LLM) agent plays a crucial role in ensuring that the generated content is suitable for children. The following outlines the iterative process involved in this integration:

**Workflow Overview**

1. Initial Content Generation: The LLM agent generates a draft story based on an input prompt. This draft is produced using the pre-trained language model, which incorporates various themes and narratives designed for children’s literature.
2. Classification Step: Once the initial draft is generated, it is passed to the classifier model. The classifier’s role is to evaluate the suitability of the content (Algorithm 3). It analyzes the title and story text to determine whether it meets the criteria for appropriateness for children.
3. Feedback Loop: After the classifier processes the generated content, it returns a suitability assessment back to the LLM agent:
  - If the content is marked as suitable: The process concludes, and the finalized story is ready for use.
  - If the content is marked as unsuitable: The classifier provides feedback regarding the specific elements that contributed to the classification. This feedback may include suggestions for adjustments or the highlighting of inappropriate themes.
4. Content Revision: The LLM agent takes the feedback from the classifier and revises the story accordingly (Algorithm 4). This may involve the following:
  - Altering phrases or themes that were flagged as unsuitable.
  - Enhancing character dialogues or descriptions to align better with child-friendly standards.
5. Iterative Process: Steps 2 through 4 are repeated iteratively until the classifier model determines that the story is suitable for children. This iterative refinement allows for an effective tightening of the narrative to ensure it meets the intended standards.
6. Final Approval: Once the classifier marks the story as suitable, the final version is saved, ready for presentation or further processing.

**Advantages of the Integrated Approach**

- Dynamic Adjustment: The iterative nature of the process allows for real-time adjustments to be made to the content, ensuring that each draft can be fine-tuned based on the classifier’s insights.
- Quality Assurance: By embedding the classifier model within the LLM framework, the overall quality and appropriateness of the generated content can be systematically enhanced.
- Child-Centric Focus: This approach prioritizes the cognitive and emotional needs of young audiences, reinforcing the commitment to producing literature that is both engaging and safe for children.

**Algorithm 3** Classify Generated Story By Sentence.
  1:**Input:** generated_story, bert_classifier  2:**Output:** sentence_classification_results (list of Suitable/Unsuitable)  3:Begin  4:Split generated_story into individual sentences  5:Initialize sentence_classification_results as an empty list  6:**for** each sentence in generated_story **do**  7:    Preprocess the sentence  8:       Tokenize the sentence text  9:       Convert the sentence to the input format required by BERT10:    Feed the preprocessed sentence into the fine-tuned BERT Classifier11:    Perform classification12:       Obtain prediction probabilities for “suitable” and “unsuitable”13:       Set threshold for determining suitability (e.g., 0.5 probability)14:    **if** prediction > threshold for “suitable” **then**15:        Append “Suitable” to sentence_classification_results16:    **else**17:        Append “Unsuitable” to sentence_classification_results18:    **end if**19:
**end for**
20:Return sentence_classification_results21:End


**Algorithm 4** Reframe Unsuitable Sentences With LLM.
  1:**Input:** generated_story, sentence_classification_results, LLM_model, classifier  2:**Output:** reframed_story  3:Begin  4:Split generated_story into individual sentences  5:Initialize reframed_story as an empty list  6:**for** each sentence and its corresponding classification result in generated_story **do**  7:    **if** classification_result == “Unsuitable” **then**  8:        Pass the unsuitable sentence to the LLM for reframing  9:           Provide context about child-appropriate content in the prompt10:           Example prompt: “Reframe the following sentence to make it suitable for children: <unsuitable_sentence>”11:        Capture the LLM’s reframed sentence as modified_sentence12:        Append modified_sentence to reframed_story13:    **else**14:        Append the original sentence to reframed_story15:    **end if**16:
**end for**
17:Combine reframed_story sentences back into a full story18:Feed reframed_story into the BERT Classifier for classification19:Initialize suitable_story as False20:**while** suitable_story == False **do**21:    Run the BERT Classifier on reframed_story to classify sentences22:    **if** all sentences are classified as “Suitable" **then**23:        Set suitable_story = True24:    **else**25:        Repeat steps 4 to 7 for any new unsuitable sentences26:    **end if**27:
**end while**
28:Return reframed_story (where all sentences are classified as suitable)29:End


## 4. Experimental Procedure

### 4.1. Environment Setup

The experiments were conducted in a controlled computational environment equipped with a robust setup to facilitate the training and evaluation of the models. We utilized a machine with an NVIDIA Quadro P5000 GPU, which provided the necessary computational power for handling deep learning tasks efficiently.

The primary deep learning framework employed for this study was PyTorch (version 2.4.1+cu124), optimized for CUDA 12.4, chosen for its dynamic computation graph capabilities and ease of use in model development. Additionally, we utilized TensorFlow (version 2.8.0) for additional model experimentation and evaluation, providing a robust framework for building and training machine learning models. The integration of pre-trained language models and the implementation of the LLM agent were facilitated through the Hugging Face Transformers library (version 4.44.2).

The environment was further optimized with CUDA version 12.6 and cuDNN version 8.9.7 to enhance GPU acceleration during training. For data preprocessing and manipulation, we relied on NumPy (version 1.26.4) and Pandas (version 2.2.2). The entire experimental setup was managed within a Python 3.9.18 environment, ensuring compatibility with all libraries and dependencies required for the successful execution of the experiments.

### 4.2. Data Preprocessing

Data preprocessing is an essential step in preparing both datasets for training the models. This phase ensures that the input data are clean, well structured, and conducive to effective analysis. The preprocessing was carried out in two main parts: one for fine-tuning the LLM and another for the classifier model.

#### 4.2.1. Dataset for LLM Fine-Tuning

- Data Cleaning: The dataset used for fine-tuning the LLM, comprising 500 children’s stories, was meticulously examined for inconsistencies, duplicates, and irrelevant entries. Any incomplete or malformed stories were removed to maintain the integrity of the dataset.
- Text Normalization: All text entries were converted to lowercase, and special characters, punctuation, and extraneous whitespace were eliminated. This standardization facilitates better tokenization and embedding representation.
- Tokenization: Each story was tokenized into individual words or subword units using the Hugging Face Transformers library’s tokenizer. This process converts the textual data into a format suitable for model input.
- Prompt Generation: Prompts were created for each story to guide the LLM during the generation phase. These prompts included essential elements, such as character names and key themes, extracted from the stories.
- Splitting the Dataset: The dataset was divided into training and validation sets using an 80:20 split ratio. This division allows for the evaluation of the LLM performance on unseen data.

#### 4.2.2. Dataset for Classifier Model

- Label Encoding: The stories designated for the classifier model were labeled as “suitable” or “unsuitable” based on their content. This binary classification scheme was converted into numerical labels (0 for unsuitable and 1 for suitable) to facilitate training.
- Data Cleaning: Similar to the LLM dataset, this dataset underwent thorough cleaning to remove duplicates and ensure that all stories met the necessary quality standards.
- Feature Extraction: Relevant features were extracted from the stories, such as story length, word count, and lexical diversity. These features were combined with BERT embeddings to create a comprehensive feature set for the classifier model.
- Scaling Features: The custom features for the classifier were standardized using the StandardScaler from the scikit-learn library. This scaling process ensures that all features contribute equally to the training, promoting faster convergence.

Through these preprocessing steps, both datasets were transformed into clean, structured formats suitable for their respective model training processes. This thorough preparation is crucial for ensuring high-quality inputs, which contribute to the overall effectiveness of the LLM agent and the classifier model in generating and evaluating child-friendly narratives.

### 4.3. Model Configuration

The model configuration phase involved selecting, initializing, and fine-tuning appropriate architectures for both the Large Language Model (LLM) and the classifier model. This configuration ensures that the models are tailored to meet the specific requirements of the tasks at hand: generating child-friendly stories and classifying them as suitable or unsuitable.

**Figure 11:**
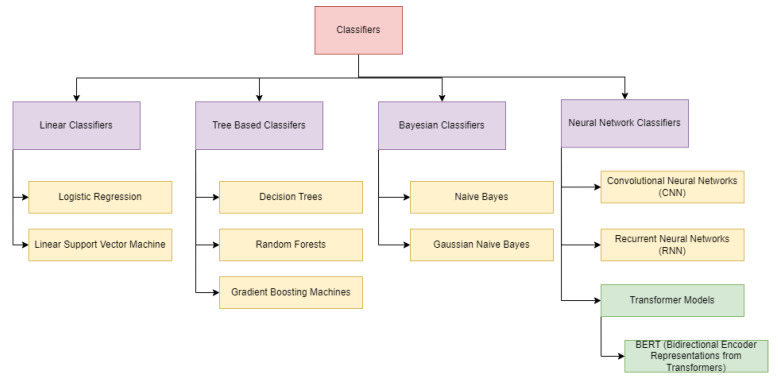
Types of classifiers.

1. LLM Configuration
  - Model Selection: We utilized several advanced generative models, specifically LLaMA 2, Mistral-7B, and Zephyr-7B-Beta, to enhance the capability of generating contextually relevant and child-friendly stories. These models were chosen for their strong performance in natural language generation tasks.
  - Fine-Tuning Process: The fine-tuning of these models was conducted using a Supervised Fine-Tuning (SFT) approach, utilizing a custom dataset comprising 515 child-friendly stories. The primary goal was to minimize the discrepancy between the model-generated text and the reference stories, focusing on fluency, coherence, and appropriateness for young audiences.
  - Training Setup: The fine-tuning process was carried out using the SFT Trainer. Given the substantial number of parameters in these models, computational resources were a concern. To address this, Low-Rank Adaptation (LoRA) was employed, which permitted updates to specific parts of the models’ attention layers without retraining the entire network, thereby reducing memory usage and computational costs while maintaining high performance.
  - Training Dataset: Each story in the custom dataset was associated with a prompt and a title. The dataset was tokenized using each model’s pre-trained tokenizer and resized to accommodate the unique vocabulary of the child-friendly dataset, ensuring proper tokenization and interpretation during training.
  - Gradient Checkpointing: To further manage memory consumption, gradient checkpointing was implemented, allowing the recomputation of intermediate activations during backpropagation. This approach enabled the models to handle longer sequences without exceeding memory limits.
  - Training Arguments:
    – Batch Size: Set to 1 per device due to memory constraints.
    – Learning Rate: A fixed learning rate of $1\times 10^{-5}$ was utilized to ensure stable convergence.
    – Gradient Accumulation Steps: Set to 4 to accumulate gradients over multiple steps, effectively addressing the small batch size limitation.
    – Evaluation Strategy: Each model was evaluated after each epoch on a validation set to monitor performance and avoid overfitting.
    – Number of Epochs: All models underwent fine-tuning for 6 epochs, with early stopping based on validation loss.
  - Loss Function: The Cross-Entropy Loss function was employed during fine-tuning for all models, measuring the divergence between the predicted and actual token distributions. By minimizing this loss, the models were guided to generate text closely resembling the reference child-friendly stories.
  - Optimizer and Learning Rate Scheduler: The AdamW optimizer was chosen for fine-tuning, decoupling weight decay from the gradient update process for improved effectiveness with large models. A cosine learning rate schedule with warm-up steps was implemented, gradually increasing the learning rate during the initial training phases followed by a slow decay, which helped prevent large fluctuations in optimization.
2. Classifier Model Configuration
  - Model Selection: The classifier model (Figure 11) is based on the BERT architecture, specifically utilizing the BERT Base variant. The BERT bidirectional attention mechanism effectively captures contextual relationships within the text, making it well suited for classification tasks, especially in understanding the language for generating child-friendly content.
  - Pre-trained Weights: The model is initialized with pre-trained weights from the Hugging Face model hub. This leverages the knowledge acquired during pre-training, allowing the classifier to benefit from vast amounts of language data, thus enhancing its performance on the specific task of classifying stories as suitable or unsuitable for children.
  - Classifier Layer: A custom classification layer is added on top of the BERT architecture. This layer consists of a fully connected neural network with two output units representing the classes “suitable” and “unsuitable”. A dropout layer is incorporated to mitigate overfitting, followed by a linear layer that produces logits, which are then passed through a softmax activation function to produce class probabilities.
  - Training Parameters: The classifier is trained using the Cross-Entropy Loss function to measure the difference between the predicted probabilities and the actual class labels. The AdamW optimizer is employed with a learning rate of $2\times 10^{-5}$, while a batch size of 8 is utilized due to memory constraints. The model undergoes training for 3 epochs, with gradient accumulation to optimize performance effectively while managing memory limitations. Each epoch involves monitoring performance on a validation set to prevent overfitting.
3. Iterative Feedback Loop: An iterative feedback mechanism was implemented, allowing the classifier model to interact with the LLM agent. After generating a story, the classifier assessed its suitability, providing feedback that guides further generation. This loop continued until the classifier marked the story as suitable, ensuring that the final output aligned with the child-friendly criteria. Through careful model configuration and fine-tuning processes, we established a robust framework for both the LLM and classifier, enhancing their ability to generate and evaluate narratives appropriate for young audiences. This configuration served as the backbone for the experimental processes that follow.
4. Training Process The training process for LLaMA 2, Mistral-7B, and Zephyr-7B-Beta models was meticulously designed to adapt these large-scale generative models for the specific task of generating child-friendly stories. This process involved several key steps to ensure that the models would produce high-quality, age-appropriate content.
  (a) Loading Pre-trained Models: The training began by loading the pre-trained versions of LLaMA 2, Mistral-7B, and Zephyr-7B-Beta. This step was crucial, as it allowed the models to utilize the extensive knowledge they acquired during their initial training on diverse text corpora, providing a strong foundation for further fine-tuning.
  (b) Tokenization and Data Preparation: The custom dataset of 515 child-friendly stories was tokenized using the respective pre-trained tokenizers for each model. This involved resizing the tokenizers to accommodate the unique vocabulary of the dataset, ensuring that the stories were properly tokenized and interpreted during the training process. Each story was paired with a corresponding prompt and title to enhance contextual understanding.
  (c) Implementing Low-Rank Adaptation (LoRA): To facilitate efficient fine-tuning while minimizing memory consumption, Low-Rank Adaptation (LoRA) was employed. This technique specifically targeted the attention layers of the models, allowing certain parameters to be updated without the need to retrain the entire network. This approach significantly reduced both computational costs and memory requirements, enabling effective model adaptation.
  (d) Gradient Checkpointing: To further manage memory usage, gradient checkpointing was enabled. This technique reduced memory consumption by storing only a subset of intermediate activations during forward propagation and recomputing the necessary activations during backpropagation. This allowed the models to handle longer sequences while staying within memory limits.
  (e) Training Configuration: The training was set up with a batch size of 1 per device, given the large model sizes and memory constraints. The learning rate was fixed at $1\times 10^{-5}$, and the models were trained for a total of 6 epochs, with early stopping based on validation loss to prevent overfitting. Gradient accumulation was utilized, allowing gradients to be accumulated over 4 steps, effectively addressing the challenges posed by small batch sizes.
  (f) Loss Function and Optimization: Cross-Entropy Loss was used as the loss function during training, guiding the models to minimize the divergence between the generated text and the reference child-friendly stories. The AdamW optimizer, coupled with a cosine learning rate schedule, facilitated stable convergence and effective optimization of the models.
  (g) Model Evaluation: Throughout the training process, the models were periodically evaluated on a validation dataset to monitor performance. This evaluation included assessing fluency, coherence, and suitability of the generated text. By tracking the performance metrics and employing early stopping, the training process ensured that the models remained focused on generating high-quality child-friendly stories. By leveraging techniques such as LoRA and gradient checkpointing, the training process for LLaMA 2, Mistral-7B, and Zephyr-7B-Beta was conducted efficiently, resulting in models capable of generating appropriate and engaging content for children.
5. Classifier Integration: The integration of a classifier model is a crucial step in ensuring that the generated stories align with child-friendly content standards. This integration involves leveraging a pre-trained BERT-based classifier to evaluate and filter the outputs from the generative models—LLaMA 2, Mistral-7B, and Zephyr-7B-Beta. The classifier’s role is to determine whether a generated story is suitable or unsuitable for children, allowing for an added layer of content validation.
  (a) Classifier Architecture: The classifier is built upon the BERT architecture, specifically utilizing the BERT Base variant, known for its bidirectional attention mechanism. This design enables the model to effectively capture the contextual relationships within the generated text, making it well suited for classification tasks. A custom classification layer is added on top of the BERT model, consisting of a fully connected neural network with two output units corresponding to the classes “suitable” and “unsuitable” for children.
  (b) Pre-training and Fine-tuning: To enhance the classifier’s performance, it is initialized with pre-trained weights from the Hugging Face model hub. This initialization allows the model to benefit from knowledge acquired during extensive pre-training on diverse text datasets. Subsequently, the classifier is fine-tuned using a curated dataset of labeled stories, which includes examples of both suitable and unsuitable content. This fine-tuning process involves training the model on this labeled data, allowing it to learn the context that differentiate child-friendly stories from those that may contain inappropriate themes.
  (c) Training Process: The training of the classifier involves utilizing the Cross-Entropy Loss function, which quantifies the difference between the predicted classifications and the actual labels. The Adam optimizer is employed with a learning rate of $2\times 10^{-5}$, and the model is trained for a predefined number of epochs. A batch size of 8 is used to ensure effective learning while managing computational resources. The classifier’s training is closely monitored through validation metrics, ensuring that it generalizes well to unseen data.
  (d) Integration with Generative Models: Once trained, the classifier is integrated into the workflow of the generative models. After a story is generated by LLaMA 2, Mistral-7B, or Zephyr-7B-Beta, the output text is passed through the classifier. The model evaluates the story, producing probabilities for each class—suitable or unsuitable. Based on a defined threshold, the classifier determines whether the generated story meets the necessary standards for child-friendly content.
  (e) Feedback Loop and Continuous Improvement: The integration of the classifier creates a feedback loop in the content generation process. Stories that are flagged as unsuitable can be analyzed to understand the reasons for the classification, allowing for iterative improvements in both the generative models and the classification criteria. This continuous improvement process is essential for refining the quality of generated content, ensuring it remains appropriate for children.
  (f) Deployment and Scalability: The classifier model, once integrated and tested, is deployed alongside the generative models in a production environment. Its scalability ensures that as the volume of generated stories increases, the classifier can efficiently evaluate them in real-time, providing immediate feedback and validation. In summary, the integration of a BERT-based classifier into the story generation pipeline not only enhances the safety and appropriateness of the content but also ensures that the generated stories are aligned with the intended audience’s needs. This approach establishes a robust framework for generating engaging and suitable narratives for children.

## 5. Results and Evaluation

### 5.1. ROUGE-1 (Unigram Overlap)

ROUGE-1 measures (Equation (7)) the overlap of unigrams (single words) between the generated text and the reference text. It captures the lexical accuracy at the word level. The ROUGE-1 score can be expressed as follows:

The fine-tuning significantly improves ROUGE-1 (Table 3) scores across all models, indicating that the training process helps the models better capture individual words from the reference text. Mistral-7B exhibits the most significant gain, jumping from 0.3185 to 0.4785 after fine-tuning. This large improvement highlights that Mistral-7B has learned to generate more accurate word matches with the reference text.

In comparison, Zephyr-7B-Beta starts off stronger in the zero-shot setting (0.3908), but its improvement after fine-tuning is less pronounced than Mistral-7B, going from 0.3908 to 0.4466. For the reframed classifier output, the scores drop slightly across the models, indicating that while the classifier improves the story’s suitability, it compromises word-level overlap slightly. The drop is modest for Mistral-7B (from 0.4785 to 0.4487), showing that the classifier may have pruned some high-frequency but less meaningful words for generating more suitable content.

### 5.2. ROUGE-2 (Bigram Overlap)

ROUGE-2 measures (Equation (6)) the overlap of bigrams (two consecutive words) between the generated and reference texts. It evaluates the model’s ability to capture word pairs and contextual word relationships.

The ROUGE-2 score can be expressed as follows:

(6) $$\mathrm{ROUGE-2}=\frac{\mathrm{Number}\;\mathrm{of}\;\mathrm{overlapping}\;\mathrm{bigrams}}{\mathrm{Total}\;\mathrm{bigrams}\;\mathrm{in}\;\mathrm{reference}}$$

The Mistral-7B model exhibits the most dramatic improvement in ROUGE-2 (0.0597 to 0.1532) after fine-tuning (Table 3), demonstrating that it learned to capture not only individual words but also their contextual relationships (word pairs) effectively. Zephyr-7B-Beta, despite having a higher zero-shot score (0.1147), shows minimal gains in bigram overlap post-fine-tuning (0.1138), suggesting its performance improvement in contextual relationships is limited compared to Mistral-7B.

(7) $$\mathrm{ROUGE-1}=\frac{\mathrm{Number}\;\mathrm{of}\;\mathrm{overlapping}\;\mathrm{unigrams}}{\mathrm{Total}\;\mathrm{unigrams}\;\mathrm{in}\;\mathrm{reference}}$$

The classifier-reframed results show a decrease in ROUGE-2 scores for all models. For Mistral-7B, it drops from 0.1532 to 0.1113, indicating that while the classifier refines the story content, it reduces the exact bigram overlap, likely prioritizing fluency or suitability over strict adherence to reference word pairs.

### 5.3. ROUGE-L (Longest Common Subsequence)

ROUGE-L measures (Equation (8)) the longest common subsequence (LCS) between the generated text and the reference. This metric emphasizes the preservation of word order and coherent structure.

The ROUGE-L score can be expressed as follows:

(8) $$\mathrm{ROUGE-L}=\frac{\mathrm{Length}\;\mathrm{of}\;\mathrm{Longest}\;\mathrm{Common}\;\mathrm{Subsequence}}{\mathrm{Total}\;\mathrm{words}\;\mathrm{in}\;\mathrm{reference}}$$

Mistral-7B shows significant gains in ROUGE-L after fine-tuning (Table 3), improving from 0.1930 to 0.2715, suggesting better preservation of the reference structure and sentence order. This indicates that Mistral-7B learns to generate text sequences that are more aligned with the reference, which is critical for story generation tasks.

In the classifier-reframed setting, all models show a slight drop in ROUGE-L, with Mistral-7B dropping from 0.2715 to 0.2186. This reflects a slight compromise in maintaining long sequences, likely due to the classifier restructuring or removing certain words to improve the suitability of the generated story.

### 5.4. METEOR (Harmonic Mean of Precision and Recall)

METEOR evaluates (Equation (9)) both precision and recall with additional factors like stemming and synonym matching. It gives more weight to recall, rewarding models for generating a higher number of relevant words while penalizing fragmentation.

The METEOR score can be expressed as follows:

(9) $$\mathrm{METEOR}=\frac{10\times \mathrm{Precision}\times \mathrm{Recall}}{\mathrm{Recall}+9\times \mathrm{Precision}}$$

The METEOR scores improve significantly post-fine-tuning (Table 3), with Mistral-7B achieving the highest score (0.4455). This reflects its ability to generate a higher number of relevant and semantically similar words, while also maintaining a good balance between precision and recall. Zephyr-7B-Beta also performs well in this metric, indicating its strength in generating semantically aligned outputs. However, the classifier-reframed models experience a slight decrease in METEOR scores, particularly for LLaMA-7B (from 0.3184 to 0.2669), as the classifier modifies the generated content to improve its appropriateness for children, often at the cost of fragmenting sentences.

### 5.5. BERT Score (Semantic Similarity of Embeddings)

The BERT Score (Equation (10)) evaluates the cosine similarity of word embeddings between the generated text and the reference, measuring the deep semantic similarity rather than surface-level word matches.

The BERT Score can be expressed as follows:

(10) $$\mathrm{BERT}\;\mathrm{Score}=\frac{\sum \mathrm{cosine}\;\mathrm{similarities}\;\mathrm{of}\;\mathrm{word}\;\mathrm{embeddings}}{\mathrm{Total}\;\mathrm{number}\;\mathrm{of}\;\mathrm{words}}$$

The BERT Score highlights the semantic improvement across all models, with Mistral-7B achieving a score of 0.8971 after fine-tuning (Table 3), showing excellent semantic alignment with the reference text. Zephyr-7B-Beta also scores highly but shows slightly lower improvement post fine-tuning.

After the classifier reframe, the BERT Score decreases slightly, with LLaMA-7B dropping from 0.8659 to 0.8332. This indicates that while the generated content is adjusted for better suitability, some semantic richness may be lost, particularly for models like LLaMA-7B.

Mistral-7B consistently outperforms other models after fine-tuning, showing significant improvements in all metrics, especially in ROUGE-2, ROUGE-L, and METEOR. This suggests that fine-tuning helps this model capture both the content and structure of the reference text more effectively than others. Zephyr-7B-Beta starts off strong in the zero-shot scenario but shows diminishing returns after fine-tuning, suggesting its base model has already learned some useful patterns.

Zephyr-7B-Beta, as a baseline, demonstrates superior performance in the initial evaluation due to its pre-trained configuration being particularly adept at handling general-purpose text generation tasks. Its architecture and training corpus may have aligned well with the general linguistic patterns and themes in the dataset prior to fine-tuning. However, its limitations became apparent when subjected to domain-specific fine-tuning for generating and categorizing children’s stories. This indicates that Zephyr might have overfit or failed to adapt effectively to the requirements of children’s literature during fine-tuning.

In contrast, Mistral-7B, though initially underperforming compared to Zephyr-7B-Beta, shows significant improvements post-fine-tuning. The architecture of Mistral, particularly its ability to learn from contextual embeddings and adapt to domain-specific training, allows it to better capture the subtleties required for evaluating the suitability of children’s stories. Its fine-tuning process effectively aligns with the task-specific objectives, leveraging its capacity to incorporate fine-grained modifications without overfitting.

These observations suggest that Mistral’s pre-training and fine-tuning compatibility enable it to excel in the task, while Zephyr’s performance post-fine-tuning highlights potential inefficiencies in adapting to domain-specific tasks despite its strong baseline results.

The classifier-reframed outputs tend to lower the lexical overlap (ROUGE-1, ROUGE-2) and semantic similarity (BERT Score), but these changes likely reflect efforts to make the content more appropriate for children. The trade-off is evident in the slightly reduced precision but better suitability for the target audience.

### 5.6. Precision-Classifier Model

Precision measures (Equation (11)) the accuracy of the positive predictions made by the model. It calculates how many of the predicted positive cases are true positives.

The precision can be expressed as follows:

(11) $$\mathrm{Precision}=\frac{\mathrm{True}\;\mathrm{Positives}\;\left(\mathrm{TP}\right)}{\mathrm{True}\;\mathrm{Positives}\;\left(\mathrm{TP}\right)+\mathrm{False}\;\mathrm{Positives}\;\left(\mathrm{FP}\right)}$$

In the proposed system using BERT Classifier, the precision for “Unsuitable” stories is significantly higher (0.95) than that for “Suitable” stories (0.73). This indicates that the BERT model is more adept at correctly predicting unsuitable stories while sometimes misclassifying suitable ones. The macro average of 0.84 suggests that precision is fairly well balanced between the classes, while the weighted average of 0.92 reflects overall strong precision, with the model leaning towards better performance in detecting “Unsuitable” content.

BERT–Random Forest Hybrid: The hybrid model improves slightly in precision for the “Suitable” class (0.75) but performs worse for the “Unsuitable“ class (0.90). This suggests that while the hybrid approach can help in some areas, BERT alone is more precise in predicting unsuitable content. The Random Forest Classifier has lower precision (0.60) for “Suitable” stories and performs comparably to BERT in “Unsuitable” predictions (0.92). This confirms that BERT outperforms Random Forest, especially for suitable content.

### 5.7. Recall-Classifier Model

Recall measures (Equation (12)) the ability of the model to identify all relevant cases, especially how well it captures the true positives.

The recall can be expressed as follows:

(12) $$\mathrm{Recall}=\frac{\mathrm{True}\;\mathrm{Positives}\;\left(\mathrm{TP}\right)}{\mathrm{True}\;\mathrm{Positives}\;\left(\mathrm{TP}\right)+\mathrm{False}\;\mathrm{Negatives}\;\left(\mathrm{FN}\right)}$$

**Figure 12:**
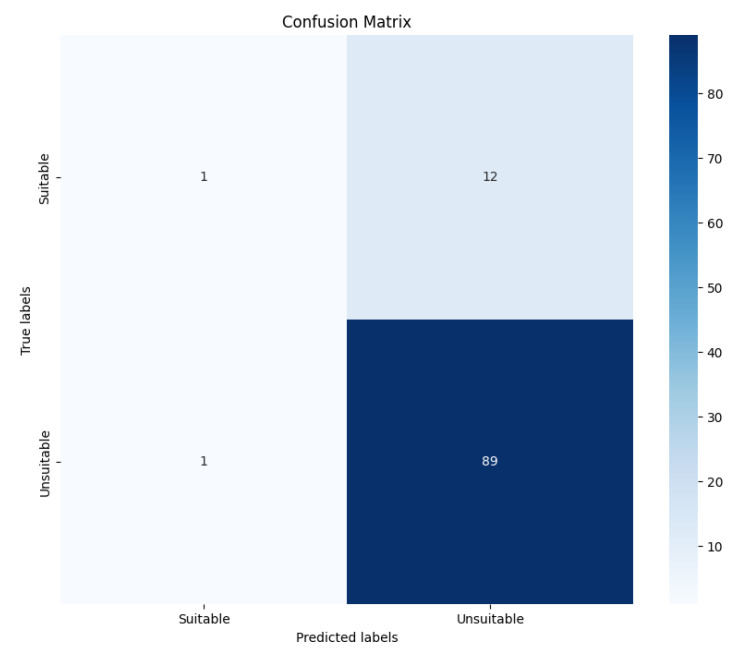
Confusion matrix BERT Classifier.

The confusion matrix of the BERT Classifier is visualized in Figure 12.

In the proposed system, BERT shows excellent recall for “Unsuitable” stories (0.97), meaning that it rarely misses these cases. However, the recall for “Suitable” stories is relatively low (0.62), suggesting that the model sometimes fails to capture all suitable content. The macro average of 0.79 indicates that the recall performance differs between classes, while the weighted average of 0.92 shows strong overall recall.

BERT–Random Forest Hybrid: The hybrid model’s recall for “Suitable” stories drops significantly (0.23), indicating that it struggles to capture suitable content. Its recall for “Unsuitable” stories is nearly perfect (0.99), but overall, the hybrid approach performs worse than BERT alone.

Random Forest Classifier: The Random Forest model performs better than the hybrid for “Suitable” recall (0.46) but worse than BERT (0.62). Its recall for “Unsuitable” stories is similar to BERT (0.96), but the overall balance of recall across both classes remains lower.

### 5.8. F1-Score-Classifier Model

The F1-Score is the harmonic mean (Equation (13)) of precision and recall, providing a balanced metric that accounts for both false positives and false negatives. The F1-Score can be expressed as follows:

(13) $$F1\;\mathrm{Score}=\frac{2\times (\mathrm{Precision}\times \mathrm{Recall})}{\mathrm{Precision}+\mathrm{Recall}}$$

The F1-Score for “Suitable” stories is moderate (0.67), which suggests a balance between precision and recall for this class. For “Unsuitable” content, the F1-Score is high (0.96), reflecting the model’s strong overall ability to accurately detect unsuitable stories. The macro average of 0.81 and weighted average of 0.92 show that BERT performs very well overall in handling both types of content.

BERT–Random Forest Hybrid: The hybrid model shows a much lower F1-Score for “Suitable” stories (0.35), which suggests it struggles in this area. The F1-Score for “Unsuitable” content remains high (0.94), but the overall performance is weaker than BERT alone.

Random Forest Classifier: The Random Forest Classifier’s F1-Score for “Suitable” content (0.52) is higher than the hybrid but lower than that of BERT. Its F1-Score for “Unsuitable” content matches that of BERT (0.94), but its macro average (0.73) and weighted average (0.89) are lower, confirming the superiority of BERT.

### 5.9. Accuracy-Classifier Model

Accuracy measures (Equation (14)) the proportion of correct predictions (both positive and negative) out of all predictions made by the model.

The accuracy can be expressed as follows:

(14) $$\mathrm{Accuracy}=\frac{\mathrm{True}\;\mathrm{Positives}\;\left(\mathrm{TP}\right)+\mathrm{True}\;\mathrm{Negatives}\;\left(\mathrm{TN}\right)}{\mathrm{Total}\;\mathrm{Samples}}$$

In the proposed system, the BERT Classifier achieves an accuracy of 0.92, meaning it correctly predicts 92% of the stories. This high accuracy reflects the model’s overall robustness, particularly in predicting unsuitable content.

BERT–Random Forest Hybrid: The hybrid model’s accuracy is slightly lower at 0.89, suggesting that integrating Random Forest does not provide significant improvements and in fact reduces accuracy.

Random Forest Classifier: The Random Forest Classifier also achieves an accuracy of 0.89, similar to the hybrid model, but still lower than that of the BERT Classifier.

In the proposed system, the BERT Classifier consistently outperforms both the BERT–Random Forest Hybrid and Random Forest Classifier in most metrics, particularly in precision and recall for the “Suitable” class. While both alternative models show some strengths, particularly in identifying “Unsuitable” content, neither performs as well as BERT overall. The BERT Classifier maintains a high level of performance across all key metrics, making it the best-suited model for the system.

## 6. Results and Discussion

The results obtained from the various evaluation metrics provide valuable insights into the performance of the proposed system, including the BERT Classifier, compared to other models like the BERT–Random Forest Hybrid and the Random Forest Classifier. This discussion explores the findings from key metrics, ROUGE, METEOR, BERT Score, Precision, Recall, and F1, and how these results contribute to the overall performance of the system designed to generate children’s stories while ensuring that unsuitable or inappropriate content is accurately filtered.

### 6.1. ROUGE, METEOR, and BERT Score Analysis

This section describes the score analysis of metrics such as ROUGE, METEOR, and BERT Score, which evaluate the performance of the models in terms of lexical overlap, semantic alignment, and contextual coherence, respectively. These metrics provide a detailed assessment of the generated stories’ quality and relevance.

#### 6.1.1. ROUGE (Recall-Oriented Understudy for Gisting Evaluation)

ROUGE measures the overlap between the generated stories and reference texts, emphasizing recall. Across the fine-tuned models, there is a noticeable improvement over the base models (zero-shot performance), especially in ROUGE-1, ROUGE-2, and ROUGE-L, which represent word, bigram, and longest common subsequence overlaps, respectively.

For example, in the Mistral-7B model, ROUGE-1 is increased from 0.3185 (zero-shot) to 0.4785 after fine-tuning. This demonstrates that the fine-tuned model captures a significantly higher proportion of relevant content from the reference stories. Similarly, the ROUGE-2 and ROUGE-L metrics are also improved, highlighting the increased coherence and fluency of the generated stories. The fine-tuning process helps the model generate output that aligns more closely with human-written stories in terms of content and structure.

#### 6.1.2. METEOR (Metric for Evaluation of Translation with Explicit ORdering)

METEOR evaluates how well the model generates text by considering the semantic content, stemming, and synonymy, thus providing a broader assessment of story quality. The Mistral-7B model, for instance, shows a noticeable improvement in METEOR, rising from 0.2918 (zero-shot) to 0.4455 after fine-tuning. This demonstrates that fine-tuning helps the model generate semantically richer stories, which are more faithful to the meaning of the reference texts. This metric shows that fine-tuning does not just improve lexical overlap but also improves the preservation of meaning, which is crucial for story generation.

#### 6.1.3. BERT Score

BERT Score measures the semantic similarity between the generated text and reference text by comparing contextual embeddings. This metric is particularly useful for assessing how well the meaning of the generated stories aligns with the intended narratives. After fine-tuning, the Mistral-7B BERT Score rises from 0.8521 to 0.8992, showing that the model’s generated stories become closer to the reference texts in terms of semantic content.

The overall improvement in ROUGE, METEOR, and BERT Score metrics after fine-tuning suggests that the models are able to generate stories that not only capture more content overlap but are also semantically richer and more aligned with human-written narratives. This combination ensures that the generated stories are not only contextually accurate but also meaningful, providing a more engaging reading experience for children.

### 6.2. BERT Classifier Performance

The BERT Classifier plays a critical role in filtering generated stories, ensuring unsuitable content is effectively removed. It outperforms both the BERT–Random Forest Hybrid and Random Forest models across most evaluation metrics, particularly in identifying unsuitable stories.

The classifier achieves a precision of 0.95 and a recall of 0.97 for identifying unsuitable content, resulting in an impressive F1-Score of 0.96. These values (Table 4) indicate that the BERT Classifier is highly reliable in detecting and filtering out inappropriate or unsuitable stories for children. The model makes precise predictions while maintaining a high recall rate, meaning very few unsuitable stories are missed by the classifier.

For suitable content, the precision is 0.73, and the recall is 0.62, indicating that the model is slightly less effective at predicting suitable stories. While the system excels in removing inappropriate content, it occasionally misclassifies suitable stories as unsuitable. This reflects a conservative approach designed to prioritize content safety for children, ensuring that no inappropriate material is generated, even if it means losing some suitable content in the process. The overall accuracy of 0.92 demonstrates that the BERT Classifier is performing well across both classes, maintaining a high standard of story selection.

### 6.3. Comparison with Hybrid and Random Forest Models

When compared to the BERT–Random Forest Hybrid (Table 5) and Random Forest models (Table 6), the BERT Classifier (Table 4) consistently demonstrates superior performance, particularly in classifying unsuitable content. The hybrid model shows poor recall for suitable stories (0.23), leading to a low F1-Score of 0.35, which indicates that the combination of BERT and Random Forest does not improve the performance for suitable content classification. While the hybrid model maintains a high precision for unsuitable stories (0.90), its overall accuracy (0.89) and precision–recall balance are not as effective as those of the BERT-only model.

The Random Forest Classifier also lags behind, with a lower precision (0.60) and recall (0.46) for suitable stories. Though it performs well in detecting unsuitable content (F1-Score of 0.94), it introduces a higher number of false positives for suitable stories. This suggests that while Random Forest is good at detecting undesirable content, it struggles to balance precision and recall for suitable stories.

### 6.4. Insights from Macro and Weighted Averages

The macro average provides an overview of performance across both suitable and unsuitable content, without considering class imbalance. For the BERT Classifier, the macro average precision (0.84), recall (0.79), and F1-Score (0.81) show a balanced but slightly biased performance, where the classifier is more effective at identifying unsuitable content than suitable stories. The weighted average, which accounts for the imbalance between suitable and unsuitable classes, offers a more accurate representation of the classifier’s performance in a real-world setting. With a weighted average precision and recall of 0.92, the BERT Classifier shows its strength in handling class imbalances effectively, ensuring that the system remains robust even when unsuitable stories make up a smaller portion of the dataset. This indicates that the BERT Classifier can manage both content safety and quality control, which is crucial for children’s story generation.

### 6.5. Impact of Fine-Tuning and Classifier Reframing

The fine-tuning of the language model significantly improves the ROUGE, METEOR, and BERT Score metrics, leading to more coherent and meaningful story generation. After integrating the BERT Classifier, the overall system precision in filtering unsuitable content rises further, ensuring that the generated stories are safe and suitable for children. While some suitable content is lost due to the classifier’s conservative approach, the system achieves its primary goal of preventing inappropriate content.

The BERT Classifier, combined with a fine-tuned generative model, provides a strong, reliable system for generating high-quality, safe stories for children. Future improvements can focus on improving recall for suitable content while maintaining the classifier’s high precision for unsuitable stories.

### 6.6. Comparison with Existing Models

The proposed methodology demonstrates superior performance compared to existing methods across various metrics (Table 7). For instance, when evaluated using the BERT Score, the proposed methodology achieves a value of 89.92, surpassing the CLICK model presented by Li Dandan et al. [34], which scored 78.2. Similarly, in Multi-Modal Story Generation, Kim Juntae et al.’s [35] method recorded a BERT Score of 22.2, significantly lower than the proposed methodology’s 89.92. In terms of METEOR scores, the proposed methodology outperforms Parag Jain et al.’s [36] SEQ2SEQ model, which achieved 10.30, by attaining a score of 40.98. Lastly, while Feng Yi et al.’s [37] Mistral-7B model achieved a commendable BERT Score of 89.8, the proposed approach slightly exceeds it with a score of 89.92. This consistent performance improvement across metrics highlights the robustness and efficacy of the proposed methodology.

### 6.7. Manual Evaluation

To evaluate the quality of the stories generated by the models, a manual evaluation was conducted involving six PhD scholars from a state university. All scholars were postgraduates with expertise in content evaluation. They were asked to rate each generated story on a scale from 1 to 5, where 1 indicates the lowest and 5 the highest. The stories were evaluated based on the following five key criteria:

- Language Simplicity and Clarity: This criterion assesses how easily the language can be understood by children. A rating of 1 reflects a high level of complexity, while a rating of 5 signifies clear and accessible language.
- Suitability for Children: This evaluates the appropriateness of the story for children, ensuring it avoids any inappropriate themes. A rating of 5 indicates complete appropriateness, while a 1 would reflect significant issues with suitability.
- Deceptiveness: This measures the accuracy and truthfulness of the content but with an inverted scale. A rating of 1 indicates that the story is less deceptive (accurate and truthful), while a 5 indicates that the story is more deceptive (misleading or false).
- Story Structure and Pacing: Evaluators assess the organization and flow of the story. A rating of 1 reflects poor structure and pacing, while a 5 indicates a well-organized and engaging narrative.
- Creativity and Originality: This criterion assesses the originality and creativity of the story. A rating of 1 indicates a lack of creativity, while a 5 reflects a highly original and inventive story.

Once the manual evaluations were completed, the ratings were analyzed to assess the consistency of the evaluations across the different evaluators. To quantify the level of agreement, the Inter-Annotator Agreement (IAA) was calculated using the Kappa score. The calculated Kappa score was 0.61, which falls within the substantial agreement range (0.6 to 0.8), indicating a high level of consistency and reliability in the evaluations.

This thorough evaluation process, supported by the Kappa score, ensured that the generated stories were assessed accurately across multiple dimensions, contributing to a robust analysis of the models’ ability to produce child-friendly content.

### 6.8. Case Study

In this case study, we examine the process of refining a children’s story to ensure its suitability for children while preserving its core themes of kindness and generosity. The study involves an input prompt, followed by a model-generated story, and a reframed version. The goal is to understand how storytelling can be adjusted in terms of content, tone, and complexity to meet the needs of children, focusing on aspects such as moral lessons, appropriateness, and accessibility. This case study illustrates the importance of refining narratives to make them not only engaging but also ethically sound for their intended audience.

1. **Input Prompt**
  Write a short children’s story about two siblings who embark on a journey to find a magical flower that spreads love and joy. Describe how their actions transform their town and teach a lesson about kindness and generosity.
2. **Model-Generated Story**
  A long time ago, there lived an old poet, a thoroughly kind old poet. He had two children: a little boy and a little girl. The little boy and the little girl were twins, and they were very good children, and they loved their old poet father very much. One day, the old poet said to them, “My dearest children, I have a special task for you. I need you to go on a journey to find a very rare and special flower that grows only in a far-off land. This flower is called the Cupid’s Blossom, and it is said to have the power to bring true love to those who possess it. But be warned, it is said that whoever finds the flower will also attract the attention of vengeful spirits, who will stop at nothing to take it from them. I know that you are both brave and kind, and I believe that you can complete this task and bring back the flower to me. Are you willing to undertake this journey?” The little boy and the little girl looked at each other and nodded eagerly. “We will do our best, father,” they said. The old poet gave them a map and a small bag of gold coins to help them on their journey. He also gave them a special amulet that would protect them from harm. The next day, the little boy and the little girl set out on their journey. They followed the map and traveled through beautiful landscapes and dangerous territories. But along the way, they encountered dark, ominous creatures lurking in the shadows, whose glowing eyes seemed to follow their every move. After a long and difficult journey, they finally reached the land where the Cupid’s Blossom grew. The flower was hidden in a secret glade surrounded by tall, ancient trees. The little boy and the little girl searched carefully and finally found the flower. They carefully picked the flower and placed it in their bag. As they were about to leave, they heard a soft, melodious voice calling to them. They turned around and saw a beautiful, winged figure with a bow and arrow. It was Cupid, the god of love. Cupid thanked them for finding the flower and said that he had been watching over them on their journey. He warned them that love could be both a blessing and a curse, and those who misuse the flower would be doomed to suffer the consequences. The little boy and the little girl thanked Cupid and promised to use the flower wisely. They said goodbye and began their journey home. As they traveled back, they thought about the importance of true love and how it could bring joy and happiness to others. They decided that when they returned home, they would use the Cupid’s Blossom to help others find love. When they finally arrived home, the old poet was overjoyed to see them. He thanked them for their bravery and kindness and praised them for their successful journey. The little boy and the little girl gave the Cupid’s Blossom to their father, and he placed it in a special vase where it could be seen and admired by all. From that day on, the old poet and his children lived in peace and happiness, knowing that they had helped bring true love to others. The Cupid’s Blossom became a symbol of love and kindness in their town, and people came from far and wide to see it. The old poet continued to write poetry about love and kindness, and his children grew up to be kind and thoughtful individuals. They remembered the lessons they had learned on their journey and carried them with them throughout their lives. And so, the story of the old poet and his children’s journey to find the Cupid’s Blossom became a beloved tale of bravery, kindness, and true love. It reminded everyone of the importance of these qualities and how they could bring joy and happiness to others.
3. **Reframed Story: Suitable for Children**
  A little boy and girl once went to search for a rare flower, which was said to bring great joy and love to those who had it. After a long journey, they found the flower and they took it home to their parents. When they gave the flower to their father, he was overjoyed and he used it to make a potion that would bring love and joy to anyone who drank it. The little boy and girl shared the potion with everyone in their town, and soon, everyone was happy and filled with love. The town became a peaceful and joyful place, and the little boy and girl were praised for their kindness. Their parents were also very proud of them, and they knew that they had done something special and important. The story of the little boy and girl’s journey and their gift of love became a beloved tale in their town, and it reminded everyone of the power of kindness and the joy that can be brought to others through simple acts of generosity. The town continued to be a place of peace and joy, and the little boy and girl grew up to be kind and thoughtful individuals, always remembering the lesson of love and joy that they had learned on their journey.
4. **Analysis of the Transformation**
  The transformation from the original story to the reframed version illustrates several key changes:
  (a) Tone Adjustment: The original story includes elements of danger, such as vengeful spirits and warnings about misusing the flower, which are removed in the reframed version. The focus is shifted entirely to the positive aspects of the story—joy, love, and generosity.
  (b) Simplification: The complex narrative involving Cupid and the curses surrounding the flower is omitted. Instead, the reframed version streamlines the plot to focus on the siblings’ positive actions and their impact on their community.
  (c) Moral Emphasis: Both versions of the story emphasize kindness and generosity. However, the reframed version places more direct emphasis on how the siblings’ actions transformed their community, making the lesson more immediate and clear to young readers.
  (d) Audience Suitability: The reframed story is more suited for children, with a simpler plot and no dark elements. It reinforces positive themes of love and kindness without the complexities of external threats, making it more approachable for a younger audience.

This case study demonstrates the ability of LLM to transform a complex narrative into a simpler, child-friendly version that retains its core values. The reframed story is an example of how LLM can adjust the tone, simplify the plot, and ensure that key themes, such as kindness and generosity, are more directly conveyed to the audience. Such transformations are particularly important when tailoring stories for children, ensuring that the narratives are both engaging and suitable for their emotional and cognitive development.

## 7. Conclusions and Future Work

This research presented a comprehensive approach to assessing the suitability of children’s stories using advanced language models and classification techniques. By employing fine-tuning strategies on state-of-the-art models, we demonstrated notable improvements in generating and classifying suitable content. The metrics derived from the evaluation of ROUGE, METEOR, and BERT Scores highlighted the efficacy of fine-tuning, with models such as Mistral-7B and Zephyr-7B-Beta outperforming the base models in generating coherent and contextually appropriate stories.

The fine-tuned models exhibited substantial enhancements, particularly in ROUGE-1 and METEOR scores, reflecting their ability to generate text that closely aligns with human-written standards while maintaining narrative coherence and relevance. Furthermore, the BERT-based classifier showcased commendable performance in precision, recall, and F1-Scores, particularly for the "unsuitable" category, indicating a robust capability to identify inappropriate content effectively.

This research also highlights the importance of model selection and training methodologies, as evidenced by the discrepancies observed between the various models, including BERT, LLaMA, Mistral, and Zephyr. The classifier’s performance validated the necessity of a tailored approach to ensure that generated stories are appropriate for children.

Future work could explore integrating more diverse datasets and leveraging additional metrics to further enhance model performance. Additionally, expanding the application of these models to other genres and languages could provide valuable insights into their adaptability and robustness in different contexts.

Recognizing the importance of extending the framework’s applicability, future work will focus on multilingual fine-tuning and classification using pre-trained models like mBERT or XLM-RoBERTa. These models provide the opportunity to address linguistic and cultural diversity effectively, allowing the framework to cater to a broader range of languages and cultural contexts. This approach will further enhance the inclusivity and global relevance of the generated content.

In conclusion, this study contributes to the growing body of research in natural language processing and its applications in content moderation, providing a framework that can be adapted for various text generation and classification tasks.

## Figures and Tables

**Table 1:** Comparison of Language Models.

Aspect	Zephyr-7B-Beta	Mistral-7B	Llama2 7B
**Model Type**	Transformer-based LLM (Auto-regressive Model)	Transformer-based LLM (Auto-regressive Model)	Transformer-based LLM (Auto-regressive Model)
**Parameter Count**	7 Billion	7 Billion	7 Billion
**Architecture**	Standard Transformer (Encoder–Decoder)	Optimized Transformer (Memory and Attention Tweaks)	Standard Transformer (Decoder-only)
**Attention Mechanism**	Multi-Head Self-Attention	Enhanced attention for speed optimization	Multi-Head Self-Attention
**Layer Normalization**	Standard Layer Normalization	Optimized Layer Normalization for faster training	Standard Layer Normalization
**Feed-Forward Networks (FFN)**	Position-wise feed-forward networks	Optimized FFN for lower memory usage	Position-wise feed-forward networks
**Tokenization**	BPE (Byte-Pair Encoding)	BPE (Byte-Pair Encoding)	SentencePiece Tokenizer (sub-word units)
**Training Objective**	Predict next token (causal language modeling)	Predict next token (causal language modeling)	Predict next token (causal language modeling)
**Special Features**	General-purpose text generation model	Optimized for faster inference and training	Strong performance across general text generation
**Intended Use Case**	General-purpose tasks (summarization, dialogue)	Real-time applications requiring low latency	General-purpose text generation (creative writing)
**Inference Speed**	Moderate	Fast	Moderate
**Memory Efficiency**	Standard	Optimized for lower memory footprint	Standard

**Table 2:** Training Parameters of Language Models.

Model	Trainable Parameters	All Parameters	Trainable Percent
LLaMA 2	311,164,928	3,811,577,856	8.16%
Mistral	352,321,536	4,110,684,160	8.57%
Zephyr	346,030,080	4,098,101,248	8.44%

**Table 3:** Performance comparison of language models.

	Model Name	Rouge-1	Rouge-2	Rouge-L	METEOR	BERT
**Base** **(Zero Shot)**	LLaMA-7B	0.2981	0.0632	0.1851	0.2799	0.8442
	Mistral-7B	0.3185	0.0597	0.1930	0.2918	0.8521
	**Zephyr-7B-Beta**	**0.3908**	**0.1147**	**0.2110**	**0.3602**	**0.8758**
**SFT-LLM Output**	LLaMA-7B	0.3617	0.0778	0.1921	0.3184	0.8698
	**Mistral-7B**	**0.4785**	**0.1532**	**0.2715**	**0.4455**	**0.8992**
	Zephyr-7B-Beta	0.4466	0.1138	0.1964	0.4154	0.8797
**Reframed-SFT-LLM** **(Classifier Output)**	LLaMA-7B	0.3105	0.0575	0.1818	0.2669	0.8554
	**Mistral-7B**	**0.4487**	**0.1113**	**0.2186**	**0.4098**	**0.8864**
	Zephyr-7B-Beta	0.4285	0.0926	0.1977	0.3963	0.8751

**Table 4:** Performance Metrics of BERT Classifier.

BERT Classifier	Precision	Recall	F1
**Suitable**	0.73	0.62	0.67
**Unsuitable**	0.95	0.97	0.96
**Accuracy**			0.92
**Macro Average**	0.84	0.79	0.81
**Weighted Average**	0.92	0.92	0.92

**Table 5:** Performance metrics of BERT–Random Forest Hybrid Classifier.

BERT–Random Forest Hybrid Classifier	Precision	Recall	F1
**Suitable**	0.75	0.23	0.35
**Unsuitable**	0.90	0.99	0.94
**Accuracy**			0.89
**Macro Average**	0.82	0.61	0.65
**Weighted Average**	0.88	0.89	0.87

**Table 6:** Performance metrics of Random Forest Classifier.

Random Forest Classifier	Precision	Recall	F1
**Suitable**	0.60	0.46	0.52
**Unsuitable**	0.92	0.96	0.94
**Accuracy**			0.89
**Macro Average**	0.76	0.71	0.73
**Weighted Average**	0.88	0.89	0.89

**Table 7:** Comparison with Existing Models.

Reference	Model/Methodology	Metric to Compare	Existing Methodology Value	Proposed Methodology Value
Li Dandan et al. [34]	CLICK	BERT Score	78.2	89.92
Kim Juntae et al. [35]	Multi-Modal Story Generation	BERT Score	22.2	89.92
Parag Jain et al. [36]	SEQ2SEQ	METEOR	10.30	40.98
Feng Yi et al. [37]	Mistral-7B	BERT Score	89.8	89.92

## Data Availability

Data will be made available on request.
